# Microstructural, Mechanical, Corrosion and Cytotoxicity Characterization of Porous Ti-Si Alloys with Pore-Forming Agent

**DOI:** 10.3390/ma13245607

**Published:** 2020-12-09

**Authors:** Andrea Školáková, Jana Körberová, Jaroslav Málek, Dana Rohanová, Eva Jablonská, Jan Pinc, Pavel Salvetr, Eva Gregorová, Pavel Novák

**Affiliations:** 1 Department of Metals and Corrosion Engineering, University of Chemistry and Technology Prague, Technická 5, 166 28 Prague 6, Czech Republic; janca.korberova@seznam.cz (J.K.); pincj@vscht.cz (J.P.); pavel.salvetr@comtesfht.cz (P.S.); Paja.Novak@vscht.cz (P.N.); 2 UJP Praha a.s., Nad Kamínkou 1345, 156 10 Prague 16, Zbraslav, Czech Republic; jardamalek@seznam.cz; 3 Department of Glass and Ceramics, University of Chemistry and Technology Prague, Technická 5, 166 28 Prague 6, Czech Republic; Dana.Rohanova@vscht.cz (D.R.); eva.gregorova@vscht.cz (E.G.); 4 Department of Biochemistry and Microbiology, University of Chemistry and Technology Prague, Technická 5, 166 28 Prague 6, Czech Republic; Eva.Jablonska@vscht.cz

**Keywords:** biomaterials, Ti-Si alloy, mechanical properties, reactive sintering

## Abstract

Titanium and its alloys belong to the group of materials used in implantology due to their biocompatibility, outstanding corrosion resistance and good mechanical properties. However, the value of Young’s modulus is too high in comparison with the human bone, which could result in the failure of implants. This problem can be overcome by creating pores in the materials, which, moreover, improves the osseointegration. Therefore, TiSi2 and TiSi2 with 20 wt.% of the pore-forming agent (PA) were prepared by reactive sintering and compared with pure titanium and titanium with the addition of various PA content in this study. For manufacturing implants (especially augmentation or spinal replacements), titanium with PA seemed to be more suitable than TiSi2 + 20 wt.% PA. In addition, titanium with 30 or 40 wt.% PA contained pores with a size allowing bone tissue ingrowth. Furthermore, Ti + 30 wt.% PA was more suitable material in terms of corrosion resistance; however, its Young’s modulus was higher than that of the human bone while Ti + 40 wt.% PA had a Young’s modulus close to the human bone.

## 1. Introduction

Even though there has been an extensive research and development of implant materials, the ideal solution has not been found yet. The crucial factors for their development are mechanical properties and corrosion, wear resistance and biocompatibility. However, one of the possible disadvantages is the stress shielding effect which can result in implants failure. This undesirable effect is associated with the different values of Young’s modulus of implants and human bone resulting in the non-uniform loading at the bone/implant interface [1]. For this reason, the porous materials have been studied extensively because they could possess Young’s modulus close to the humane bone and can solve the problem with the stress-shielding effect. Moreover, materials with porous structure also allow better osseointegration due to the ingrowth of bone tissue into implants. Moreover, the osseointegration takes place for a short time with good quality [1], and is the important property for biomaterial.

The porous biomaterials provide better fixation to the bone in contrast with dense biomaterials. The transport of body fluids through the implants is easier due to pores resulting in the ingrowth of tissue [1]. This ingrowth depends on material, the size and shape of pores, overall porosity, the character of pores system, biocompatibility and Young’s modulus. The optimal pore size varies in the range from 100 µm to 500 µm and pores must be interconnected for maintaining the vascular system [2]. The porous biomaterial possesses low Young’s modulus allowing to adjust its value to the value corresponding to human bone, which can solve the problem with stress shielding effect. The value of Young’s modulus of human cortical bone is stated as 10 to 30 GPa [3].

The porous metallic implants can be classified based on the various criteria. The first of them divides implants to the ones a porous surface and to the implants with volume porosity [2]. The second classification divides the porous implants to the open- and close-pore systems. Implants with close pores have each pore completely separated by a thin metallic wall, while the implants with open pores have the pores interconnected. The interconnected net of pores enables the penetration and the incorporation of the tissue to materials [3]. The porous materials with open pores are preferred for the processing of biomaterials.

Titanium and its alloys [4], mainly TiAl6V4 [2,5], nitinol (NiTi) [6,7,8,9], zinc, cobalt and its alloys [10,11], magnesium [12], tantalum [13,14] and stainless steel [15,16] are the most studied biomaterials. Nowadays, most of the implants are usually produced from titanium and its alloy due to their superior corrosion resistance, good mechanical properties and non-toxicity; however, the stress shielding effect occurs when titanium is applied as the implant. It is caused by their stiffness which is much higher than stiffness of bone tissue [1]. Young’s modulus of pure titanium is too high (110 GPa) in comparison with the modulus of the human bone (approximately 20 GPa) [17]. Thus, the mentioned presence of pores can hinder this unwanted effect. The titanium also possesses good biocompatibility and its alloys are bioinert [1,18]. The titanium alloys free of toxic elements show even the best biocompatibility [16]. The porous titanium is mainly suitable for the dental and orthopaedic implants although, the process of the surface treatment is still necessary to obtain bioactive surface [5]. On the contrary, the ions of vanadium and aluminum contained in the most used alloy, TiAl6V4, were considered as the components causing health issues such as neurological or enzymatic disorders or disturbers [19]. However, its releasing amount is negligible and does not cause the health issues. Nitinol, with extraordinary properties such as superelasticity and shape memory effect [6], is applied as orthodontic wires and intravascular stents [20]. Cobalt and its alloys excel in terms of fatigue strength, wear resistance and corrosion resistance as well. Hip replacements or dental implants are made of Co-Cr-Mo alloy [11]. Fractures repairs, such as bone plates, bone screws, pins or rods used solely stainless steel [16]. Many of the mentioned and listed biomaterials are designed mainly for permanents, traumatological implants or for replacements of joints. The new porous alloy Ti-Si was chosen as a potential candidate for implants specially determined for application where the ingrowth of bone tissue into implants is required. The silicon belongs to the group of biogenic elements and thus, this one should not cause undesirable reactions, such as toxicological or allergic reactions in the human body. Moreover, silicon is a biocompatible element strengthening the titanium alloys [21]. Another indisputable advantage for processing is a narrow crystallization range and good fluidity [21]. Furthermore, it was found that the number and size of pores increase with increasing content of silicon in titanium alloys [22].

As it was mentioned, one of the possibilities how to improve osseointegration is using of porous biomaterials. One of the ways to obtain porous biomaterials is the preparation of materials containing pore-forming agent (also called as space-holder) by powder metallurgy. This method comprises the mixing of powders, compaction, removing of pore-forming agent and sintering [23]. Firstly, the pore-forming agent in powder form, e.g., NaCl, TiH_2_ ZrH_2_ or NH_4_HCO_3_, is mixed with a mixture of metals whose particle sizes must be less than pore-forming agent powder. The second step of the process is producing the green bodies through the uniaxial or isostatical compression at ambient temperatures. Subsequently, the pore-forming agent is removed from the green bodies during low-temperature heat treatment or during dissolution in the solvent which generates pores (the initial stage of neck formation). After removing the pore-forming agent, the sintering at higher temperatures allows us to obtain more porous alloy due to the Kirkendall effect in the case of alloys—mainly due to the developing sinter neck growth [3,17,18,24]. It is believed that the biggest advantage is the possibility to control the mechanical properties by choice of size, shape or the amount of pore-forming agent [3].

In this work, the porous Ti and Ti-Si alloys with the pore-forming agent were prepared by Self-propagating High-temperature Synthesis (SHS). The important characteristics and properties for biomaterials applications, such pore size, Young’s modulus, corrosion resistance or cytotoxicity were studied. These alloys were studied as potential candidates for the bone supports and substitutes (e.g., augmentation).

## 2. Materials and Methods

### 2.1. Preparation of Samples

The porous titanium samples with 20, 30 and 40 wt.% of the pore-forming agent (PA), TiSi2 alloy with 20 wt.% of PA, pure titanium and TiSi2 alloy without PA were prepared by powder metallurgy. Titanium powder (particle size <45 µm, purity 99.98%, Sigma-Aldrich, St. Louis, MO, USA) and 2 wt.% of silicon (<45 µm, 99.5%, Alfa Aesar, Kandel, Germany) were mixed with the appropriate amount of PA for 10 h at 45 rpm in Turbula 3D blender. Ammonium bicarbonate NH_4_HCO_3_ was chosen as PA because its decomposition is accompanied by the formation of gaseous NH_3_ and CO_2_. Subsequently, the obtained mixture was cold-pressed at an isostatic pressure of 400 MPa. The samples with PA were decomposed thermally for 16–18 h at 100 °C. The samples TiSi2 were heated under inert atmosphere (Ar) at a heating rate of 100 °C/min in an induction furnace with a holding time of 10 min at 1100 °C, during which the samples were reactively sintered. Titanium without PA and with various contents of PA was sintered in a vacuum furnace at 800 °C for 1 h with a subsequent increase in temperature to 1300 °C, at which they were sintered for 4 h.

### 2.2. Microstructure and Phase Composition

All sintered samples were ground by sandpapers P180–P2500. Mechanical-chemical polishing was performed using colloidal suspension Eposil F (ATM GmbH, Mammelzen, Germany) mixed with hydrogen peroxide (volume ratio 1:6). Kroll’s agent (10 mL HNO_3_, 20 mL HF, 170 mL H_2_O) was used for etching which took for 2 s.

The microstructure was observed by the optical microscope Olympus PME3 with Axio Vision Rel. 4.8 software (Olympus, Tokyo, Japan). The phase composition was studied by PANalytical X’Pert Pro (Cu anode, K_α_ radiation; PANalytical, Almelo, The Netherlands) and results were analyzed using X’Pert High Score Plus software with PDF2database only in the cases TiSi2 and TiSi2 + 20 wt.% of PA to found out the kind of formed silicides.

### 2.3. Porosity

The evaluation of resulted porosity (labeled as porosity by image analysis) and pores size were performed using program Lucia 4.80 (Laboratory Imaging, Prague, Czech Republic) and the optical macrographs were used. The volume porosity (labeled as porosity by weight) was calculated according to Equation (1) expressed as:

(1) $$V_{porosity}=\;\frac{m_{0}}{m_{1}},$$

where *m*_0_ is the weight of the compacted specimen and *m*_1_ is the actual weight of the porous alloy.

### 2.4. Mechanical Properties

The Vickers microhardness was measured with a load of 100 g (HV 0.1) using device Future-Tech FM-700 (Future-Tech, Kawasaki, Japan). Furthermore, the compressive tests were performed using universal loading machine LabTest5.250SP1-VM (Labortech, Opava, Czech Republic) and three tested samples were prepared by cutting to obtain the dimensions 5 × 5 × 7.5 mm. The three-point flexural tests were performed via the same universal loading machine, but the dimensions were 5 × 5 × 25 mm.

The impulse excitation technique was applied to calculate Poisson’s ratio, Young’s modulus and shear modulus on the base of measurements of the resonant frequencies. The cylindrical samples with a height of 4 mm and a diameter of 38 mm were tested using the RFDA IMCE machine (IMCE, Genk, Belgium) where the samples were fastened through the nylon fiber. Subsequently, a small projectile tapped on the surface of tested samples and the induced vibration signals were recorded by the microphone. The acquired vibration signals were converted to the resonant frequency through Fourier transformation which enabled us to calculate the Poisson’s ratio, Young’s modulus and shear modulus.

### 2.5. Electrochemical Measurements

The cylindrical samples with a diameter of 15 mm and height of 3 mm were used for corrosion tests. These samples were ground by sandpapers P400–P2500 and, subsequently, ultrasonically cleaned in water and ethanol.

The electrochemical measurement was carried out in physiological solution (9 g/L NaCl) using a potentiostat Gamry 600 with a Gamry Instrument Framework software (Gamry Instruments, Warminster, PA, USA). A standard three-electrode set-up with a Ag/AgCl/sat. KCl electrode (ACLE) as a reference electrode and a graphite electrode as the counter electrode was used. For the determination of the corrosion rate, two methods of measurements were applied—the measurement of polarization resistance and potentiodynamic curves. In the case of polarization resistance measurement, the open-circuit potential (OCP) was stabilized for 3600 s followed by the polarization in the range from −20 to +20 mV/OCP and with scanning rate of 0.125 mV/s. The total exposed area of the sample was 1 cm^2^. The potentiodynamic curves were obtained during anodic (from −0.05 V/OCP to 1 V/OCP) polarization with scanning rate 1 mV/s. The exposed area was also 1 cm^2^.

### 2.6. In Vitro “Bioactivity” Tests

The samples were cut into plates with a height of 3.5–4 mm. Furthermore, the samples were ground by sandpapers P80–P800. The hole with a diameter of 2 mm was drilled in the middle of samples for nylon fiber. The polyethene (PE) vials with a volume of 100 mL were filled with ethanol and the samples were hung separately into vials. The vials with samples were inserted to the ultrasonic bath for 10 min. The samples were taken out and dried in air at ambient temperature for a few days.

The in vitro tests started after drying. Studied alloys (pure Ti, Ti + 40 wt.% PA, TiSi2, TiSi2 + 20 wt.% PA) were immersed in simulated body fluid (SBF) for 7 days at 37 °C in biological thermostat. The SBF solution was prepared according to the study [25]. All preparation was performed at 36.5 ± 1.5 °C and tris(hydroxymethyl)aminomethane (TRIS)+HCl was used as a buffer. Samples were hung to PE vials filled by SBF (100 mL) and pH was measured by intoLab 7110 (WTW) with a glass electrode (3 mol/L KCl) after the first, fourth and seventh day of exposure. At the same time, 1 mL of extract was removed and the quantities of ions Ca^2+^ and (PO_4_)^3−^ were determined. The same volume of extract was also removed from the SBF solution without sample at the first and last day of the experiment. This enabled us to observe the concentration of these ions without samples. Finally, samples were rinsed with distilled water and dried with flowing air at ambient temperature after the end of *immersion* tests. The evaluation of immersion tests included measurements of many crucial parameters. The first group of parameters consisted of the measurement of pH of extracts, gravimetry of samples and their surface. As it was mentioned, the values of pH were observed after the first, fourth, and seventh day. Furthermore, samples were weighed using analytical balance METTLER TOLEDO AG204 (Mettler Toledo, Amar Hill, India) before and after tests. The surfaces of studied samples were observed using an optical microscope Olympus BX51 (Olympus, Tokyo, Japan) and scanning electron microscope Tescan Vega 3 LMU (Tescan, Brno, Czech Republic) equipped with energy dispersive spectrometry OXFORD Instruments X-max EDS SDD 20 mm^2^ (Oxford Instruments, High Wycombe, UK).

The second group of parameters included the determination of Ca^2+^ and (PO_4_)^3−^ ions in SBF. The concentration of Ca^2+^ was analyzed by atomic absorption spectrometer GBC 932 plus (GBC Scientific Equipment, Dandenong, Australia) and by flame atomizers (acetylene and N_2_O). On the contrary, the concentration of (PO_4_)^3−^ ion was determined by ultraviolet-visible spectroscopy (UV-VIS) at a wavelength of 830 nm using spectrophotometer UV-2450 SHIMADZU (Shimadzu, Kyoto, Japan).

### 2.7. In Vitro Cytotoxicity Tests

The samples with the highest amount of pore-forming agent (Ti + 40 wt.% PA and TiSi2 + 20 wt.% PA) were chosen for in vitro cytotoxicity tests and Ti and TiSi2 were used as referent samples. The shape of the studied samples was cylindrical, with a diameter of 15–16 mm and height 3–4 mm. The surface was ground by sandpapers P80–P2500 and rinsed in acetone and ultrasonically cleaned in ethanol for 15 min.

First of all, samples were sterilized in sterilizer Memmert UFP 700 (Memmert GmbH, Schwabach, Germany) at 180 °C for 2 h. Thereafter, sterilized samples were transferred to MEM (Minimal Essential Medium) with 5% FBS (Fetal Bovine Serum). The reduced amount of FBS was used as recommended in the standard ISO 10993-5 [26]. The total amount of MEM + FBS solution was 4–5 mL per sample in order to reach surface-to-volume ratio of 1.25 cm/mL as recommended in the standard ISO 10993-5 [26]. The incubation of samples proceeded at the temperature of 37 °C for 24 h on an orbital shaker Infors HT (Infors HT, Bottmingen, Switzerland) with speed 120 rpm. The extracts were further used for in vitro cytotoxicity tests (elution test).

The elution in vitro cytotoxicity test was performed according to ISO 10993-5 standard [26] with murine fibroblasts L929 (ATCC^®^ CCL-1^TM^, Manassas, VA, USA) which were cultivated in MEM + 10% FBS at standard conditions. The suspension of 1 × 10^5^ cells/mL was prepared. This suspension was seeded into 96-well plates (6 repetitions per each sample). Well plates, located at the edges, were filled by MEM + 10% FBS, but they were not used for the experiment due to the different temperature and moisture. These plates were inserted into incubator Sanyo where the cells were cultivated at 37 °C for 24 h under 5% CO_2_.

**Figure 1:**
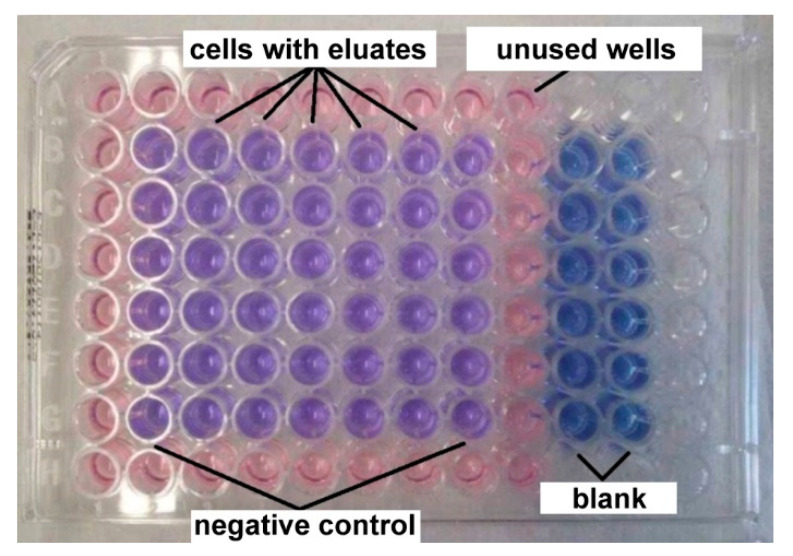
The arrangement of 96-wells plates for cytotoxicity tests.

Thereafter, murine fibroblasts were observed by optical microscope Olympus CKX41 (Olympus, Tokyo, Japan) in order to confirm their uniform growth. The medium was removed from wells and 100 µL of extracts of tested samples was added to semi confluent cell layer. The second and eight columns of plates containing only 100 µL MEM + 5% FBS served as a negative control (Figure 1).

After 24 h exposition to extracts, the metabolic activity was evaluated. The extracts were removed and wells were filled by 100 µL of MEM + 10% FBS without phenol red + resazurin (25 µg/mL). The wells, labelled as a blank, contained 100 µL of MEM + 10% FBS without phenol red + resazurin and no cells. These plates were incubated for 60 min in the incubator.

The test is based on the ability of viable cells to reduce blue resazurin to fluorescent and violet resorufin. The fluorescence of resorufin was measured using fluorometer Spectramax Minimax i3x platform Molecular devices (Molecular device, San Jose, CA, USA) at input wavelength 560 nm and output wavelength 590 nm and compared to the negative control. The metabolic activity 70% of the control was taken as a minimum value of cytocompatibility.

## 3. Results and Discussion

### 3.1. Microstructure and Phase Composition

**Figure 2:**
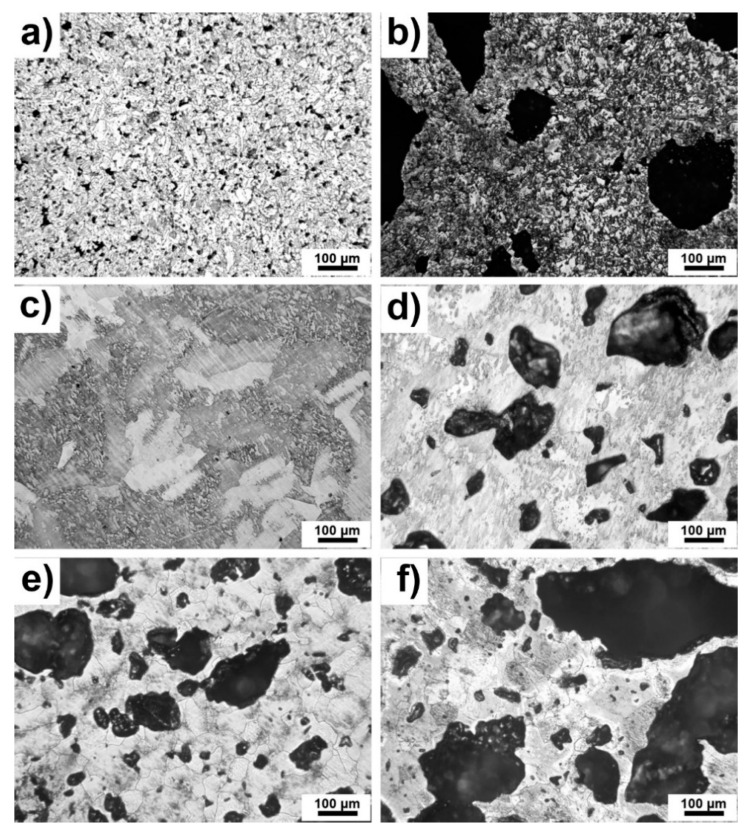
Microstructure of alloys (**a**) TiSi2; (**b**) TiSi2 + 20 wt.% PA; (**c**) Ti; (**d**) Ti + 20 wt.% PA; (**e**) Ti + 30 wt.% PA; (**f**) Ti + 40 wt.% PA.

Optical micrographs are shown in Figure 2a–f. The present pores can be observed as black spots from the smallest ones to the largest ones depending on chemical composition. It seems that pores are open and interconnected which was also observed in [27]. Residual porosity was low and caused by the reactive sintering of pure Ti. Furthermore, the obvious differences in the structure of Ti were affected by the orientation of titanium grains which were etched differently (Figure 2c). It can be seen that the pores were much larger in TiSi2 alloy than in pure Ti (Figure 2a,c), which is caused by the Kirkendall effect and by the different lattice parameters of titanium and silicon influencing the resulted porosity. In the cases of studied alloys with PA, the porosity was caused by both sintering and the decomposition of the pore-forming agent. It is obvious that the porosity evidently increased with the addition of PA. The pore-forming agent was decomposed to gaseous ammonia and carbon dioxide resulting in the formation of the pores. Moreover, in the cases of TiSi2 + 20 wt.% PA alloy (Figure 2b), pores were formed by Kirkendall effect due to unbalanced diffusivities of titanium and silicon during reactive sintering itself and by the decomposition of PA.

Table 1 listed porosity by image analysis and porosity by weight calculated from the theoretical and real weight of samples. If the samples are isotropic (the absence of a directional dependence of porosity), uniform (the absence of porosity gradients) and the location of pores is random (without periodicity of the porosity), the Delesse–Rosiwal law should be valid [28]. According to this law, the porosity by image analysis is equal to porosity by weight. This one was confirmed by the presented values shown in Table 1. Only in the case of TiSi2 alloy, the porosity by weight was significantly higher than porosity by image analysis, suggesting the non-uniform distribution of pores within the alloy.

As was expected, the porosity increased with increasing addition of PA which was already obvious from microstructure (Figure 2a–f). The porosity of TiSi2 + 20 wt.% PA was approximately twice as high as of Ti + 20 wt.% PA alloy. The explanation lies in the mentioned Kirkendall effect and the differences between titanium and silicon lattice parameters. This one explained also the porosity determined for pure Ti and TiSi2 because all processes of sintering elemental powders are susceptible to the formation of Kirkendall pores.

**Figure 3:**
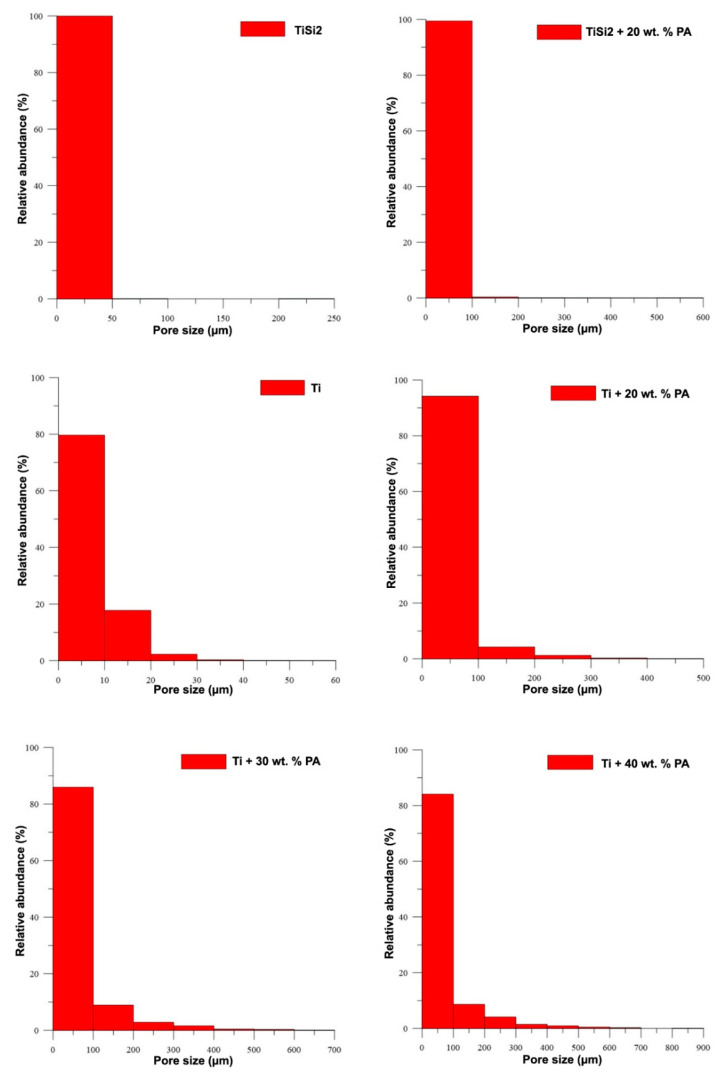
Relative representation of pore size.

The histograms shown in Figure 3 illustrate the relative representation of pore size for all studied samples. These results confirmed the increasing pore size with the amount of added PA and the beneficial addition of PA for the synthesis of porous alloys. The observed pores with the size smaller than 100 µm were found in TiSi2 + 20 wt.% PA. Only a few of pores have the size larger than 200 µm. On the contrary, titanium with 20 wt.% PA contained above 90 % of pores with size below 100 µm, 5% of pores smaller than 200 µm and 2 wt.% of pores whose dimensions did not exceed 300 µm. The rest of the observed pores have a size of approximately 500 µm. It is known that the optimal pore size varies in the range of 100–500 µm [2] for the ingrowth of bone tissue into the implants. Only the samples with pore-forming addition contained such large pores; however, almost 80% of them were smaller than 100 µm.

**Figure 4:**
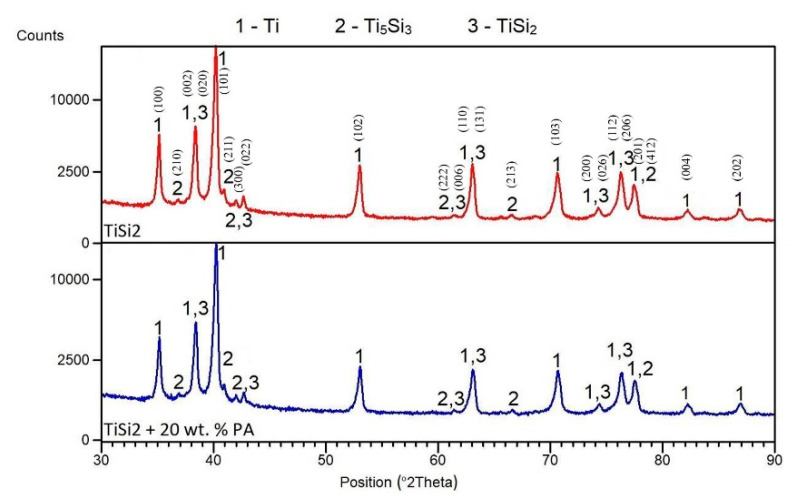
XRD patterns of TiSi2 and TiSi2 + 20 wt.% PA alloys.

The matrix was formed by hexagonal titanium and a solid solution of silicon in α-Ti, which was confirmed by X-ray analysis. X-ray diffraction was performed to identify the types of silicides formed in TiSi2 and TiSi2 + 20 wt.% PA alloys during reactive sintering. X-rays analyses revealed the presence of Ti_5_Si_3_ and TiSi_2_ silicides (Figure 4) suggesting the nonequilibrium behavior during reactive sintering. This claim was confirmed by comparison of the expected phase composition with the equilibrium phase diagram [29]. Therefore, it is obvious that Ti_5_Si_3_ phase formed due to the kinetical factors [30]. Despite the presence of the pore-forming agent, phase composition was not influenced.

**Figure 5:**
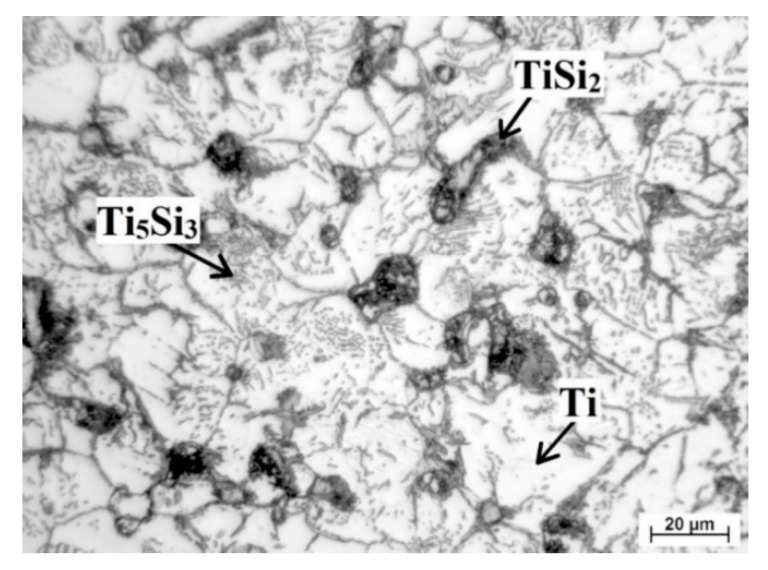
Detail of microstructure of TiSi2 alloy.

Found phases are marked in Figure 5 and was identified according to work [22], which studied the formation of silicides in porous Ti-Si alloy. Titanium formed matrix (white area) while the light grey round particles at the boundaries were Ti_5_Si_3_ phase (confirmed during EDS analysis). Silicides TiSi_2_ occurred primarily at the edges of pores. The presence of those phases is not a surprise. Trambukis et al. [31] found the phases sequence of formation during SHS, which is: TiSi_2_ → TiSi → Ti_5_Si_4_ → Ti_5_Si_3_. According to this sequence, it is clear that TiSi_2_ phase forms preferentially and it is gradually transformed to the more stable Ti_5_Si_3_ phase. Moreover, it was confirmed that Ti_5_Si_3_ phase can also form by the direct reaction between titanium and silicon [32] and it is the most thermodynamically stable phase (∆H_f_ = −579 kJ/mol) in the Ti-Si system [33]. The high surface-to-volume ratio of small titanium particles enables full contact between titanium and silicon, resulting in the formation of intermediate phase at lower temperatures and the mechanism in the initiation of the SHS reaction was the transformation of α-Ti to β-Ti, as shown in [33].

### 3.2. Mechanical Properties

**Figure 6:**
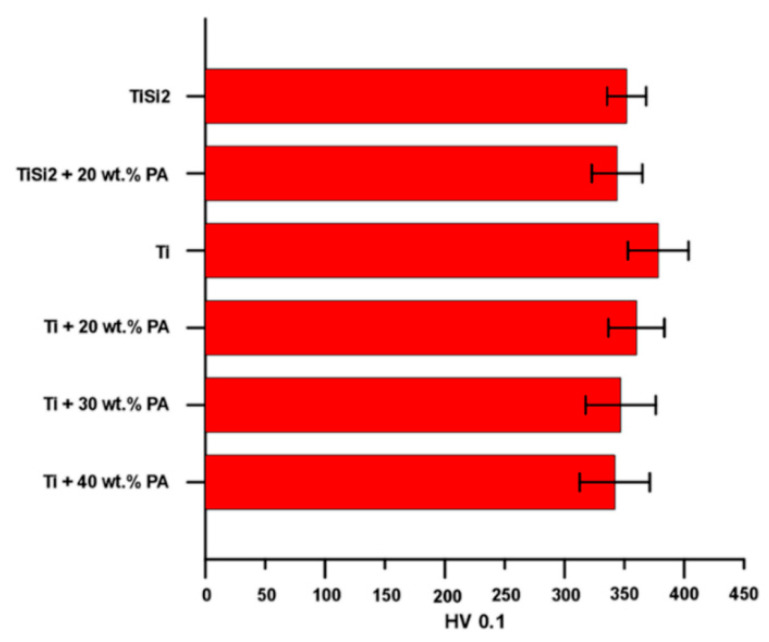
Microhardness HV 0.1 of studied alloys.

First of all, the microhardness was measured only on areas without pores and results are shown in Figure 6. In the case of TiSi2 and TiSi2 + 20 wt.% PA, the microhardness of the matrix (not silicides) was determined. The microhardness of silicides was impossible to measure because as it was shown, they appeared as thin and elongated particles close to pores (Figure 2a,b). The resulted values of microhardness were approximately similar for all tested alloys.

**Figure 7:**
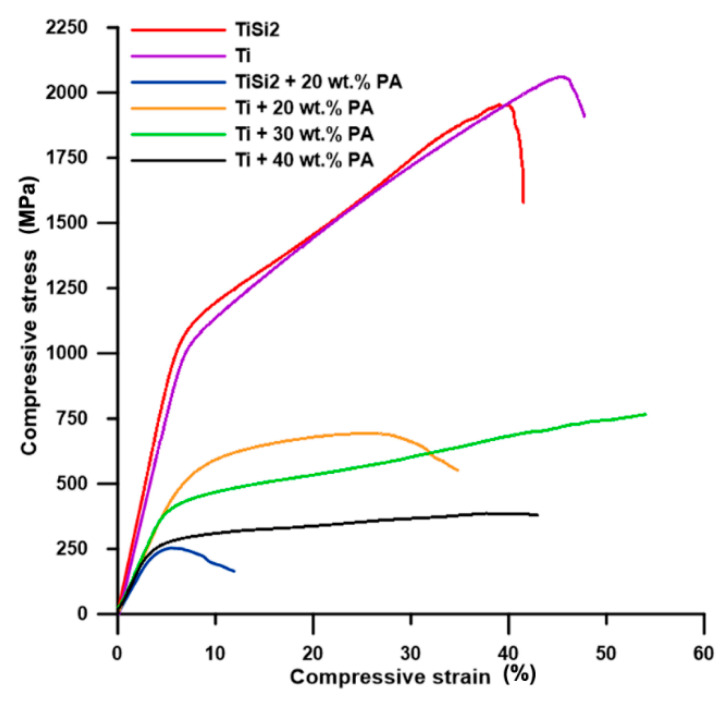
Engineering compressive stress–strain curves.

The course of compressive stress–strain curves of pure Ti and TiSi2 alloy was similar (Figure 7) and the slight decrease in compressive strength belonging to TiSi2 alloy was mainly affected by higher volume porosity. However, both samples exhibited high compressive strength (Table 2) resulting from their microstructure.

The shape of the compressive stress–strain curve of Ti + 40 wt.% PA was typical for highly porous metallic materials. The present pores absorb the applied strain which can be seen as the low increase or almost constant stress accompanied by a significant increase in compressive strain (Figure 7). Tang et al. [34] presented the deformation course of Ti with 40–70% porosity compared to deformation curve observed for Ti + 40 wt.% PA alloy in this work. The course of the deformation curve belonging to Ti + 30 wt.% PA (Figure 7) was the same. The increase in compressive strain is also obvious, together with compressive stress, which is associated with lower porosity and pore size for both alloys. In these two cases, the compressive stress–strain tests were stopped manually when the compressive stress increased significantly. Thus, the values of R_p0.2_ (yield strength) could be deducted while the values of R_m_ (ultimate compressive strength) were not listed in Table 2. All prepared alloys possessed much higher yield strength, compressive ultimate strength and higher or same the value of Young’s modulus than those of porous titanium scaffolds shown in work [1] and TiAl6V4 scaffolds presented in work [5]. Nevertheless, the values were too high for human cancellous bone (E = 0.9 GPa and R_m_ = 22 MPa) [1], but the obtained modulus generally suits the modulus for cortical bone varying in the range of 10–30 GPa [16].

The sample Ti + 20 wt.% PA exhibited low plasticity in comparison with samples containing more pores (Figure 7). However, the fracture could be observed in Ti, Ti + 20 wt.% PA, TiSi2 + 20 wt.% PA and TiSi2 alloy. The deviations of stress were observed on the stress–strain curve of TiSi2 + 20 wt.% PA during loading. It can be assumed that they are caused by the presence of silicides around pores. These silicides could hinder the absorption of loading by pores which influenced the plastic behavior of the material. The unusual large pores also caused very low R_m_ of TiSi2 + 20 wt.% PA (Table 2) suggesting the decreasing of Rp_0.2_ and E with increasing the porosity (Table 2).

**Figure 8:**
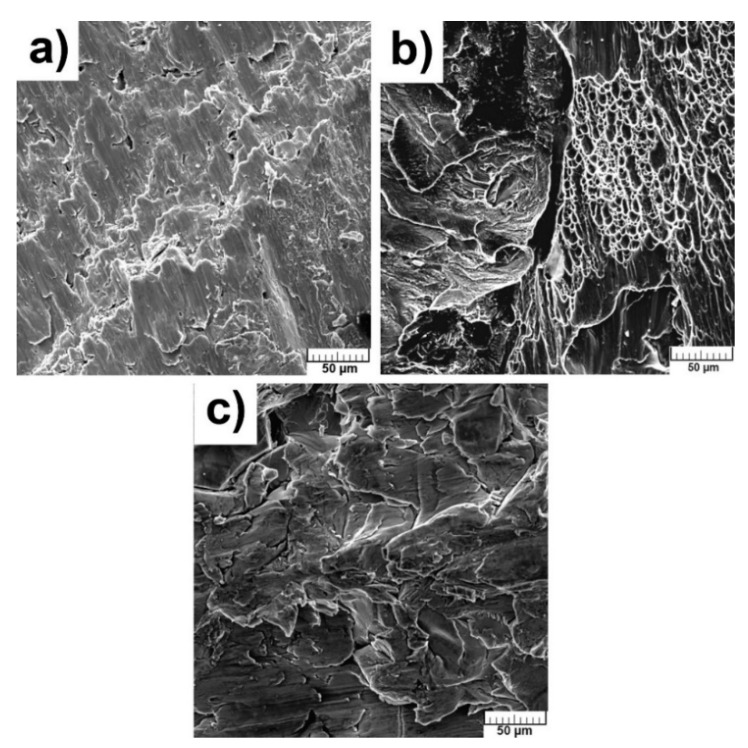
Fracture surface of alloys: (**a**) TiSi2; (**b**) Ti; (**c**) Ti + 20 wt.% PA.

The fractographic characterizations were performed after compressive stress–strain tests to reveal the mechanism of failure and morphologies are shown in Figure 8a–c. The dimples, meaning the ductile fracture, are obvious only in pure Ti (Figure 8b). The morphology of fractures of TiSi2 and Ti + 20 wt.% PA alloys did not contain dimples (Figure 8a,c) but no facets were also observed, so the cleavage fracture could not be considered. However, the courses of obtained curves can be proof of the ductile fracture of studied materials. The fracture took place diagonally suggesting the shear stress acting at the end of measurement due to dislocation slip. The grain boundaries were weakened by the presence of brittle Ti_5_Si_3_ phase in the case of TiSi2 alloy.

The results obtained by impulse excitation are shown in Table 3. The value of the Poisson number (µ) of the studied titanium sample is very close to the value of pure titanium (0.32) [35]. On the other hand, the Poisson number of TiSi2 is the lower which is probably caused by the higher volume porosity and by the presence of silicides. Table 3 also revealed that porosity has a significant effect on the value of the Poisson number. Its value decreased with the increment of porosity. The shear modulus was calculated from the Poisson number and Young modulus and results are also shown in Table 3. The calculated value of the shear modulus of studied titanium is close to the value stated for pure titanium, which is approximately 43 GPa [35]. Because the values of shear modulus are dependent on measured Poisson number and Young’s modulus, the trends between values are the same as mentioned above. The values of modulus of TiSi2, Ti, Ti + 20 wt.% PA samples are satisfied for the using as the trabecular bone replacement [36]. On the other hand, the values of the modulus of all samples were higher than those of human cancellous bone, although they fulfill the requirements to pore size and porosity (Table 1, Figure 5) for human cancellous bone (pore size of 20–1000 µm and porosity 30–95%) [18]. Obviously, Ti + 30 wt.% PA having volume porosity 35 ± 1%, Young’s modulus 47 ± 1 GPa (from compressive tests) and 9 ± 1 GPa (impulse excitation) is suited as the replacement of human cortical bones [37]. The elastic modulus of porous alloys strongly depends on the number of pores, the pore size and also the pore morphology [34] which is reflected by our results in Table 1 and Table 3, and confirmed this statement. The different values of Young’s modulus obtained by impulse excitation and from the compressive stress–strain curves are affected by the method of measurement. The samples contained the pores acting as stress concentrator and, thus, they make easier the spreading of crack. The value of Young’s modulus is deducted from the linear part of obtained compressive stress–strain curve. Meanwhile, the samples are not loaded during impulse excitation and, therefore, the present pores do not affect the mechanical properties.

**Figure 9:**
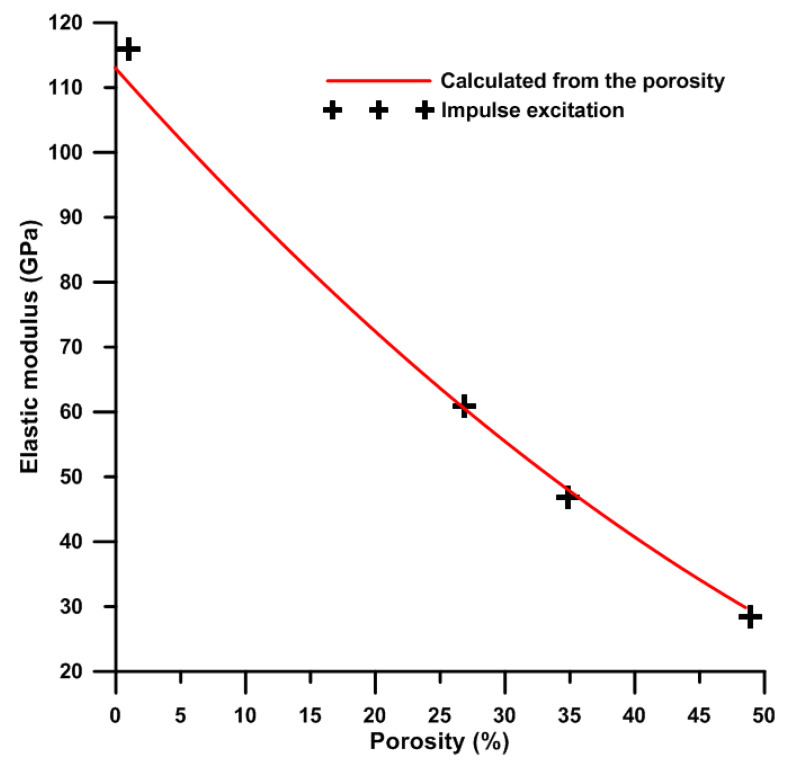
The dependence of Young’s modulus (calculated and experimental determined) on porosity of titanium.

The Young’s modulus obtained from impulse excitation was compared with the theoretical one calculated according to the Gibson–Ashby model [18] is expressed by Equation (2):

(2) $$\frac{E}{E_{0}}=k\;{\left(1-P\right)}^{2},$$

where E is Young’s modulus for porous titanium, E_0_ is Young’s modulus for compact titanium, k is the constant (equal to 1 for metals) and P is the porosity of porous titanium. The value 113 GPa [35] belonging to pure titanium without pores was considered for calculation. As can be seen, the values obtained from impulse excitation lies on the curve of theoretically calculated values (Figure 9). This means that the impulse excitation is an accurate method for the determination of the Young’s modulus, unlike the compressive stress–strain tests. Furthermore, for both of the experimental (impulse excitation) and calculated results, Young’s modulus decreases with the increasing porosity (Figure 9).

The bending strength obtained during three-point flexural tests is listed in Table 4. Besides the results of compressive stress–strain tests, the bending strength during flexural tests varied in a wide range. The reason lies in the presence of silicides and less the volume porosity. When the TiSi2 + 20 wt.% PA is compared to Ti + 40 wt.% PA with the same volume porosity, the different bending strength is affected by silicides, but increasing porosity decreased the bending strength. The bending strengths were also about 2.5–16 times lower than as-cast TiSi1 in [21], which can be explained by the presence of pores.

### 3.3. Corrosion Resistance

#### 3.3.1. Electrochemical Measurements

**Figure 10:**
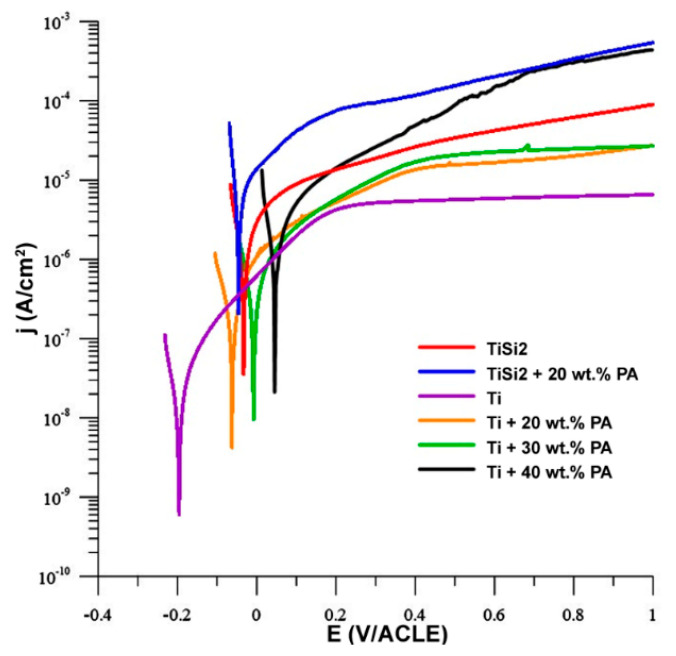
Anodic polarization in simulated body fluid (SBF).

The anodic curves of potentiodynamic measurement are shown in Figure 10 and only these curves are presented because they illustrated the oxidation of metals. The oxidation of titanium was already described systemically in [38,39]. In our work, the sample of Ti exhibited an increase in the current density in range 0.2–1 V, suggesting the passivation of the sample. In addition, the passivation could be observed in the case of Ti + 20 wt.% PA and Ti + 30 wt.% PA as well. On the contrary, the instability of the passivation layer can be found in Ti + 40 wt.% PA which resulted in the local maxima and impossibility of stabilization of current density from 0.4 V/ACLE. The alloys with Si addition were not able to passivate (Figure 10).

**Figure 11:**
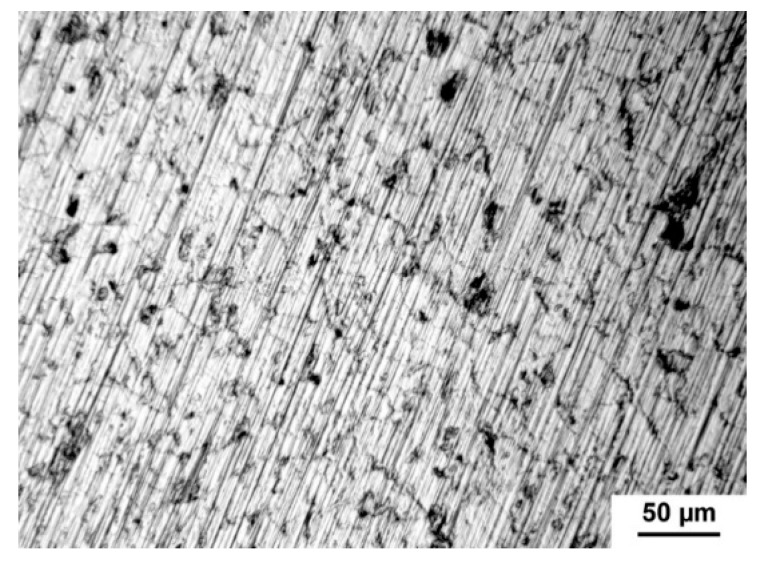
Intergranular corrosion of TiSi2 alloy.

The measured values obtained from the anodic and cathodic polarization are listed in Table 5. Tafel slopes (b_a_ and b_c_) were determined from the anodic and cathodic parts of potentiodynamic curves. Both quantities were used for the calculation of corrosion rate which was related to geometric area 1 cm^2^. However, the real area of samples depends on the size and the number of pores contacted with electrolytes and, therefore, the real area is much higher than the geometric area in the case of porous materials. It is known that the pores decrease the corrosion resistance of titanium because they increase the specific surface area its contact with the medium [34]. The present pores could also induce the localized corrosion influencing the values shown in Table 5. The corrosion rate increased with increasing porosity. The R_p_ (polarization resistance) value of studied porous alloys is significantly smaller than those obtained for the cast titanium [1] which confirmed the important effect of pores in structure. The silicon addition also affected the corrosion rate. As can be seen, TiSi2 alloy had significantly higher corrosion rate in comparison with the sample with the same volume porosity Ti + 20 wt.% PA. For this reason, this sample was studied in detail after corrosion experiments. The study of the exposed structure revealed the intergranular corrosion meaning the weakening of grain boundaries (Figure 11) due to the present of silicides. The higher corrosion rate in Ti + 40 wt.% PA than in TiSi2 + 20 wt.% PA with similar volume porosity was probably caused by the high current density obtained from cathodic curves where the reduction in environmental components took place.

#### 3.3.2. In Vitro “Bioactivity” Tests

No changes of pH measured for extracts were observed during exposure in comparison with the SBF without samples. The value of pH was approximately 7.42 at 36.5 °C. In addition, no significant changes in weight were observed (Table 6). The negative weight loss was observed in the case of TiSi2 and TiSi2 + 20 wt.% PA suggesting the dominance of corrosion of those samples over the precipitation of Ca-P phase. The slight increase in weight (Ti and Ti + 40 wt.% PA) could be caused by the precipitation of low amounts of sodium chloride on the surface whose presence was confirmed during EDS analysis.

**Figure 12:**
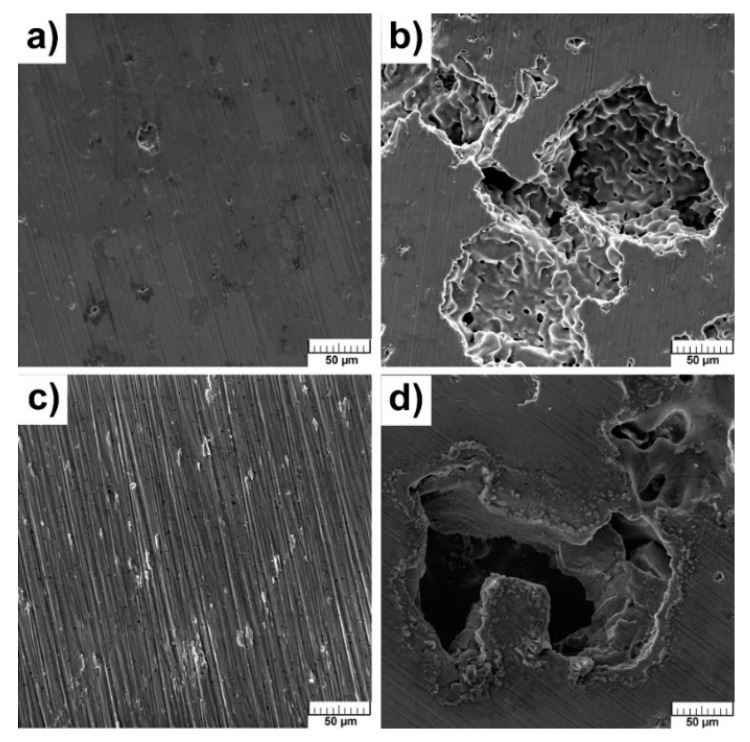
Microstructure of alloys after exposition in SBF: (**a**) TiSi2; (**b**) TiSi2 + 20 wt.% PA; (**c**) Ti; (**d**) Ti + 40 wt.% PA.

The surface of samples did not change after exposure in SBF and no hydroxyapatite, Ca_10_(PO_4_)_6_(OH)_2_, was found which could point to bioactivity. The hydroxyapatite has exceptional biocompatibility and bioactivity properties [12]. SEM analysis did not reveal the presence of Ca-P phase. The sodium chloride occurred mainly at the edges of the pores (Figure 12a–d).

**Figure 13:**
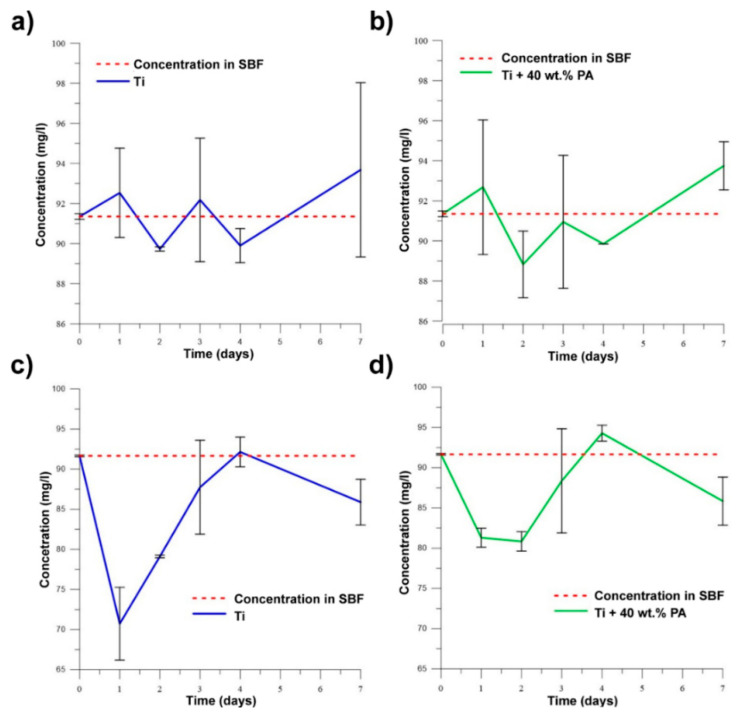
The changes in concentration of (PO_4_)^3−^ for (**a**) Ti; (**b**) Ti + 40 wt.% PA and of Ca^2+^ for (**c**) Ti; (**d**) Ti + 40 wt.% PA in SBF.

When the samples exhibit bioactive behavior, the concentration of ions (PO_4_)^3−^ and Ca^2+^ decreases in SBF due to the precipitation of the Ca-P phase. It can be assumed that alloys with Si will not be bioactive according to results of SEM analysis and pH values. Figure 13a–d clearly show that no studied samples are bioactive because the concentration of ions (PO_4_)^3−^ and Ca^2+^ increases in time. The observed declines were associated with the adsorption of ions on the surface with their subsequent release into solution. As is known, titanium is a bioinert material [18], and this work showed that the pores did not affect bioactive behavior through the absorption of ions with the subsequent precipitation of required phases. It is still necessary to solve the problem of the bioactivity of studied samples and investigate possible methods to improve it. This obstruction can be successfully resolved by the titania sol-gel coating which was tested on TiSi alloys and presented in [40].

### 3.4. In Vitro Cytotoxicity Testing

**Figure 14:**
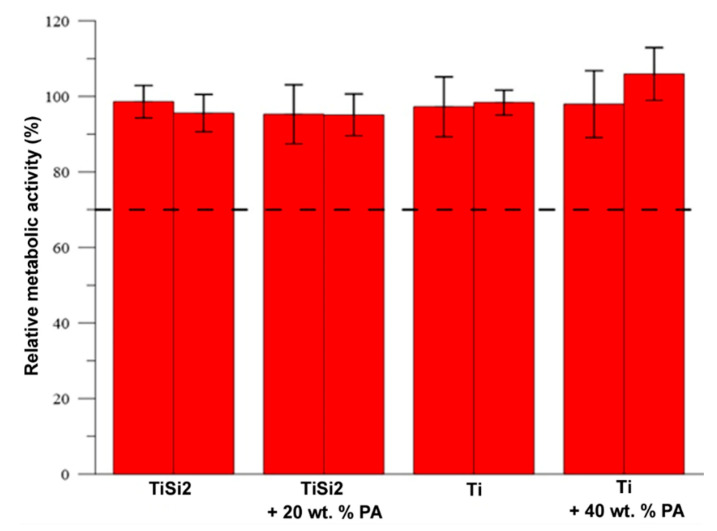
Metabolic activity of L929 cells after 24 h incubation with the extracts.

The in vitro cytotoxicity of samples TiSi2, TiSi2 + 20 wt.% Pa, Ti and Ti + 40 wt.% PA was tested according to the ISO standard. The murine fibroblasts L929 were exposed to extracts of the samples for 24 h. The materials are considered as cytocompatible (not toxic) when the metabolic activity of the cells does not decrease below 70% of the negative control, which is illustrated as the dashed line in Figure 14. As can be seen, none of the extracts caused a decrease in metabolic activity after 24 h incubation with the cells. Thus, according to the elution test, the samples can be considered as cytocompatible. Neither silicon addition nor PA implemented the cytotoxicity and, therefore, the developed materials seem promising for biomedical application.

## 4. Conclusions

The porous alloys were prepared successfully by powder metallurgy. All Ti-Si samples with pore-forming agent contained pores with the size of 100–500 µm suitable for bones ingrowth but most of them were found in Ti + PA. Titanium with PA also exhibited better mechanical characteristics and plasticity than TiSi2 + 20 wt.% PA alloy. Higher values of yield strength belong to Ti + PA in both used tests (the compression and the three-point flexural tests). However, the most important parameter for augmentation or spinal replacements is Young’s modulus. The values of the Young’s modulus of TiSi2 + 20 wt.% PA (30 ± 1 GPa) and Ti + 40 wt.% PA (28 ± 1) were close to the Young’s modulus of human cortical bone. In the case of these alloys, the volume porosity was similar—47 ± 1% (TiSi2 + 20 wt.% PA) and 49 ± 1% (Ti + 40 wt.% PA). The corrosion resistance was affected by the presence of silicides in microstructure causing the corrosion along the grain boundaries. For this reason, titanium with PA addition samples possessed better corrosion resistance. Moreover, the pores filled by electrolyte created a more aggressive environment due to the worse exchange of electrolyte. Bioactivity and cytotoxicity were not observed. The Ti + PA and Ti + 30 wt.% PA were determined as the most suitable for porous implants.

## Figures and Tables

**Table 1:** Resulted porosity.

Sample	“Porosity by Image Analysis” (%)	“Porosity by Weight” (%)
TiSi2	2 ± 1	15 ± 3
TiSi2 + 20 wt.% PA	37 ± 11	47 ± 1
Ti	1 ± 0	2 ± 1
Ti + 20 wt.% PA	24 ± 5	27 ± 1
Ti + 30 wt.% PA	31 ± 3	35 ± 1
Ti + 40 wt.% PA	46 ± 5	49 ± 1

**Table 2:** Mechanical properties obtained from compressive stress–strain curves.

Sample	R_p0.2_ (MPa)	R_m_ (MPa)	E (GPa)
TiSi2	980 ± 18	1846 ± 153	18 ± 0
TiSi2 + 20 wt.% PA	250 ± 52	291 ± 26	8 ± 0
Ti	1039 ± 31	2104 ± 61	19 ± 0
Ti + 20 wt.% PA	508 ± 13	686 ± 9	10 ± 0
Ti + 30 wt.% PA	386 ± 4	-	9 ± 1
Ti + 40 wt.% PA	233 ± 22	-	8 ± 0

**Table 3:** Mechanical properties of studied alloys obtained by impulse excitation.

Sample	µ	G (GPa)	E (GPa)
TiSi2	0.28	30 ± 1	77 ± 2
TiSi2 + 20 wt.% PA	0.26	12 ± 1	30 ± 1
Ti	0.33	44 ± 1	116 ± 4
Ti + 20 wt.% PA	0.28	24 ± 1	61 ± 1
Ti + 30 wt.% PA	0.27	18 ± 1	47 ± 1
Ti + 40 wt.% PA	0.26	11 ± 1	28 ± 1

**Table 4:** Strength of studied alloys during three-point flexural tests.

Sample	R_m_ (MPa)
TiSi2	454 ± 36
TiSi2 + 20 wt.% PA	111 ± 15
Ti	652 ± 31
Ti + 20 wt.% PA	412 ± 0
Ti + 30 wt.% PA	328 ± 21
Ti + 40 wt.% PA	252 ± 35

**Table 5:** Results of anodic and cathodic polarization.

Sample	Anodic Polarization			Cathodic Polarization			R_p_	v_corr_
	b_a_	E_corr_	j_corr_	b_c_	E_corr_	j_corr_		
			×10^−6^			×10^−6^	×10^3^	×10^−3^
	(V/dec)	(V/ACLE)	(A/cm^2^)	(V/dec)	(V/ACLE)	(A/cm^2^)	(Ω × cm^2^)	(g/cm^2^ × a)
TiSi2	0.68	−0.033	6.1	0.41	−0.020	5.4	12	36.8
TiSi2 + 20 wt.% PA	0.90	−0.046	38.1	0.41	−0.073	35.0	2	272.1
Ti	0.19	−0.196	0.1	0.17	−0.253	0.1	330	0.5
Ti + 20 wt.% PA	0.43	−0.062	1.3	0.34	−0.069	0.8	48	6.8
Ti + 30 wt.% PA	0.35	−0.008	1.4	0.54	0.093	4.0	11	33.7
Ti + 40 wt.% PA	0.37	0.045	5.3	-	0.133	114.3	1	573.5

**Table 6:** The changes in weight after 7 days in SBF solution.

Sample	Weight Change (g)
TiSi2	−0.023 ± 0.004
TiSi2 + 20 wt.% PA	−0.050 ± 0.016
Ti	0.001 ± 0.000
Ti + 40 wt.% PA	0.003 ± 0.000
